# Early verb production in Nungon

**DOI:** 10.3389/fpsyg.2023.1241447

**Published:** 2023-08-31

**Authors:** Hannah S. Sarvasy

**Affiliations:** MARCS Institute for Brain, Behaviour and Development, Western Sydney University, Penrith, NSW, Australia

**Keywords:** child language acquisition, Papuan, Nungon, verb, production, inflection, variation, development list paragraph

## Abstract

This brief research report presents a comparison of the early verb productions of four children acquiring the Papuan language Nungon as a first language. A previous case study examined only verb productions of the child TO; these are now compared with those from three other children, studied from ages 1;1–2;7 (non-dense corpus; one child, AB) and ages 2;4–2;7 (dense corpora; two children, MK and MF). Two of the most striking features of TO’s early verb productions are shown to be outliers relative to the other three children: her ‘root nominals’ stage and her delayed near future tense production. Neither of these is transparently linked to patterns in her parents’ child-directed speech. The other children also display differing strategies into language production. The dense corpus is beneficial for catching tokens of less-frequent inflections, but the frequent long recording sessions may be difficult for at least one child to tolerate.

## 1. Introduction

This report presents evidence for the early verb productions of four children, aged 1;1–2;7, learning the Papuan language Nungon in a village setting. Nungon is considered part of a Finisterre grouping of languages (Claassen and McElhanon, 1970) in the rugged region between the Finisterre and Saruwaged mountain ranges in northeastern Papua New Guinea. Typical of the extreme linguistic diversification in Papua New Guinea, Finisterre languages show a wide range of grammatical features and lexical forms (Sarvasy, n.d.), and Nungon itself is just an umbrella term for the southern six dialects of a dialect continuum; there are between 30 and 50 households that speak each dialect, or roughly 300–400 people each. The discussion here focuses on the Towet village dialect of Nungon. In this section, I give background on the Nungon verb system (section 1.1), then present characteristics of Nungon child-directed speech (CDS; section 1.2), then describe what is known of children’s early verb productions (section 1.3).

### 1.1. The Nungon verb

Nungon has a rich system of verbal inflections, described in full in the Nungon reference grammar (Sarvasy, 2017). Verbs comprise a short, 1-3-syllable root, followed by one or more suffixes that indicate tense, mood, and modality and index subject person/number. Each verb root belongs to one of six classes, determined by the forms tense and subject suffixes take after the root. Unrelated to these classes, a sub-group of transitive verbs obligatorily bear a prefix indexing person/number of their object argument (this prefix encodes singular versus non-singular number values, in contrast to the subject suffixes, which encode singular versus dual versus plural number: see Sarvasy, 2018). Aspectual distinctions are indicated through auxiliary constructions involving the lexical verb (either in root form or with an additional suffix), immediately followed by the auxiliary verb *it-* ‘exist, be’ or *to-* ‘do.’ The Nungon general verb template is in (1); here, the slot for tense is in parentheses because immediate imperatives, contrafactuals, and some other inflections lack an indication of tense.

1. (Object)-root-(tense)-subject

Example (2) includes the “H-class” verb *na-* ‘eat’; although transitive, *na-* ‘eat’ is not one of the verbs that takes obligatory object prefixes.

2. Omop    na-wa.
    Pandanus   eat-imp.1sg
    Let me eat pandanus. (MF, 2;4)

Since the verb *mon-* ‘throw’ belongs to the “C-class” of verbs, the 1sg imperative suffix is *-e* (and the final /n/ of the verb root lenites to /r/ before the vowel), as in (3):

3. To-ng   mor-e.
    sg.o.take-dep  throw-imp.1sg
    Let me throw it away. (AB, 2;7)

Example (4) includes the C-class verb *w-e-* ‘beat s.o./s.t.’, which obligatorily indexes both object and subject person/number.

4. Amna   morö   w-e–engka-t.
    Man   big   3sg.o-beat-nf-1sg
    I will beat a big man. (MF, 2;4)

Table 1 shows all Nungon verbal inflections with the verb *na-* ‘eat’ and a 1sg subject argument, as well as non-inflecting deverbal nominalizations.

**Table 1:** First person singular subject forms, verb *na-* ‘eat’.

Tenses	Remote past	*na-go-t*	I ate (yesterday or before)
	Near past	*na-wa-t*	I ate (earlier today or yesterday)
	Present	*na-ha-t*	I am eating, or I usually eat
	Near future	*na-wangka-t*	I will eat (later today), or I eat if…
	Remote future	*na-i-t-ma*	I will eat (tomorrow or beyond)
Other verbal inflections	Immediate imperative	*na-wa!*	Let me eat!
	Delayed imperative	*na-it*	Let me eat (later)
	Irrealis	*na-it*	I might eat, lest I eat
	Contrafactual	*na-hem*	I would have eaten, or should I eat?
	Dependent (same subject)	*na-ng*	Eat (within a multi-verb predicate)
	Dependent (different subject)	*na-wa*	I eating (within a multi-verb predicate)
	Medial (same subject)	*na-nga*	Eating
	Medial (different subject)	*na-wa-ya*	I eating
	Probable	*na-wang*	S/he/I may eat
Multi-verb predicate aspectual constructions	Desiderative/imminent aspect	*na-wang-na ta-a-t, to-go-t, to-t, to-wangka-t, t-i-t-ma*	I want to eat, or I am about to eat
	Habitual	*na-ng it-ta-t, it-do-t, i-i-t-ma*	I always eat/ate/will eat
	Continuous	*na-nga it-ta-t, it-do-t, e-t, e-ngka-t, i-i-t-ma, ir-a, e–e-ya,* etc.	I am eating/I was eating, I will be eating, etc.
	Continuous habitual	*na-nga ir = it-ta-t, ir = it-do-t, ir = i-i-t-ma, ir = e–e-ya,* etc.	I always am eating, I used to be eating, I will always be eating, etc.
	Inferred imperfective	*na-nga ta-ga-t*	I seem to be eating
	Completive	*na-ng = dup ta-a-t, to-go-t, to-t, to-wangka-t, t-i-t-ma, to-em, to-wa,* etc.	I am eating it all up, I ate it all up, I will eat it all up, etc.
Nominalizations	Participle	*na-ng-gang*	Eating
	Nominalization	*na-k-na-k*	Eating
	Iterative	*na-ng-na-ng*	Eating

In Nungon, five tenses are distinguished: a remote past tense, used for hesternal (‘yesterday’) and prior time frames; a near past tense, used for both hodiernal (earlier today) and hesternal events; a present tense, used for the ‘gnomic’ (general) present, currently in-progress events, and recently concluded events with present relevance; a hodiernal near future tense, used for ‘later today,’ but also with some applications as a general and conditional future tense; and a remote future tense, used for ‘tomorrow’ and beyond.

The Nungon immediate and delayed imperative paradigms include forms for first and third persons, along with the cross-linguistically prototypical (or ‘canonical’: Aikhenvald, 2010) second person imperative forms. As in other Nungon inflectional paradigms, the second and third person imperative forms are different for singular number but show syncretism in dual and plural number, such that second-person dual imperative has the same form as the third person dual imperative, and the same is true for the second and third person plural imperatives. That is, *na-warun!* is used for both ‘you two eat!’ and ‘let the two of them eat!’, and *na-warut!* is used for both ‘you (three or more) eat!’ and ‘let them (three or more) eat!’ This may be taken as evidence that second person imperatives should be analyzed as forming part of an imperative paradigm with the other person inflections.

Nungon is a clause chaining language (see Sarvasy, 2015, 2017, 2020 and Sarvasy and Choi, 2020a,b, n.d.). This means that sentences can include multiple ‘chained’ clauses, of which only the last one bears indication of tense or mood, and of which all other clausal predicates bear indication of whether the following subject will be the same or different as the current one (‘switch-reference’ marking). An example of a three-clause chain from the child MK at age 2;4 is in (5).

5. Mama   ongo-nga bap(iya) kö-i-ya
    Mother.bt go-mv.ss paper   sg.o.raise-ds.2sg-mv
    aa-wa.
    sg.o.see-imp.1sg
    Mama, you going, raising the paper, let me see it. (MK, 2;4)

In (5), MK displays appropriate use of switch-reference marking: ‘going’ shares a subject (2sg) with the following clause, so is marked for ‘same-subject’ (ss), while ‘raising’ has a different subject from the following clause (2sg vs. 1sg), so is marked for ‘different-subject’ (DS). The fact that the entire sentence is framed in the imperative mood is only indicated on the final verb, ‘see.’ Nungon also allows for multi-verb predicates, such that each clause may have more than one verb root in its predicate; in multi-verb predicates, only the last verb root bears inflections.

### 1.2. Special features of Nungon child-directed speech

Nungon child-directed speech (CDS) involves:

- Pitch modifications (but no vowel hyper-articulation: Sarvasy et al., 2022);
- Optional consonant modifications, especially the replacement of rhotics with laterals or palatal glides, and the replacement of syllable- and word-final /k/ with glottal stops (Sarvasy, 2017);
- A baby-talk or nursery lexicon that includes alternative forms for nouns, like *ede* ‘ghost, picture’ for adult *dogu*, and uninflecting verb alternatives, like *dai* ‘sleep’ and *purik* ‘turn’ for adult *duo-* ‘sleep’ and *iwan-* ‘turn’ (Sarvasy, 2017, 2019);
- A morpho-syntactic alteration: what would be directly-inflecting verbs in normal adult-directed speech (ADS), such as *obö-wa-k* ‘break-np-3sg,’ ‘it broke,’ are expressed by nominalizing the main, lexical, verb, then supporting this with an inflecting auxiliary verb *to-* ‘do’: *obö-k to-k* ‘break-nmz do-np.3sg,’ ‘it did breaking’.

The ‘do’ auxiliary construction of (d) could be said to simplify the learning task in terms of morphology by confining inflection to the same verb, *to-* ‘do,’ so the child does not have to use different inflectional suffixes for different verb classes (since nominalized verbs do not inflect). At the same time, though, it arguably makes the utterance syntactically more complex, by expanding what would be a single verb into two separate words. This construction is attested for a wide range of lexical verbs in CDS, including intransitive and stative verbs. An example directed at AB from his father when AB was 1;5 is in (6):

6. Father: Obö-k  to-wangka-k;na-mo-kt   o-i.
    break-nmz do-nf.sg-3sg  1sg.o-give-nmzdo-imp.2sg
    It will do breaking; do giving it to me. (AB 1;5)

In normal ADS, this would not occur; the verbs *obö-* and *na-mo-* ‘give me’ would normally inflect directly, and it would be nonsensical to add an auxiliary *to-* ‘do’; the usual ADS version of (6) is in (7):

7. Obö-wangka-k;    na-mo-hi.
    break-nf.sg-3sg   1sg.o-give-imp.2sg
    It will break; give it to me.

In (7), the singular near future tense suffix *-wangka-* immediately follows the verb root *obö-* ‘break,’ with no auxiliary *to-* ‘do,’ and the same is true of the 2sg immediate imperative suffix *-hi* on the verb *na-mo-* ‘give me.’

Even the stative verb *it-* ‘exist, stay, be’ can be expanded in this way in CDS, as seen in (8), from AB’s father when AB was 2;7:

8. Father: Ng-ondo  it     to-rangka-mok.
       prox-near be.nmz  do-nf.du-1du
       The two of us will do staying here. (AB 2;7)

This sounds nonsensical in a non-CDS context; the usual way of producing this in ADS is in (9):

9. Ng-ondo   it-dangka-mok.
    prox-near  be-nf.du-1du
    The two of us will stay here.

### 1.3. Previous work on acquisition of Nungon verbal inflections

Although the early acquisition of verbal inflections by children is of prime theoretical interest (Bittner et al., 2003; Aguado-Orea and Pine, 2015), acquisition of Nungon verbal inflections was previously only investigated as a case study of one child, TO, studied from age 2;1 to 3;3 (Sarvasy, 2019). TO showed early use of the 2sg imperative inflection, present tense inflection, and 1pl imperative inflection. She showed noticeably delayed use of the near future tense inflection, months after she produced tokens of all other tense inflections and both imperative types. This delay was especially remarkable because the near future tense was consistently used more frequently by TO’s parents in the recording sessions than either the remote future or the near past tense. In Sarvasy (2019, 2022), I speculated that her late production of the near future tense could be: (a) a sampling artifact; (b) due to the higher morphophonological complexity of the near future tense; (c) due to limited contexts for children to use the near future tense; or (d) due to the near future tense’s polyfunctionality as a hodiernal future and as a conditional future.

TO also exhibited a pattern in which she used a nominalized verb as an optional default verb form, in contexts where an adult would use an inflected verb. This is essentially producing a ‘do’ auxiliary construction as in CDS without the ‘do’ auxiliary: just the lexical verb in nominalized form, without a supporting inflecting verb. TO’s use of the nominalized verb in this way (here, with a ‘specifier’ suffix that she often adds to it) is shown in example (10), an exchange between TO, age 2;7, and her mother, centering on unscrewing the audio recorder from its small tripod.

10. Mother: Ök,   ngo-go   usi-ha-t,
        ij    prox-adv  extract-pres.sg-1sg
        papa = ho       e-nga.
        paternal.uncle.bt = foc  come-mv.ss
        Yes, I unscrew (it) like this, Uncle having come.
  Child:  Mama    uti-c = ma-o.
         mother.bt   extract-nmz = spec-top
         Mama unscrewing (it). [Describing her mother’s having unscrewed the recorder; adult form would be *usi-ha-rok* ‘you (sg.) unscrew (it).’]
           …
  Child:  Au   uti-c = ma     mama.
         other extract-nmz = spec mother.bt
         Unscrewing (it) again, Mama. [Asking to unscrew it again; adult form would be *usi-wa*, the 1sg imperative form].
  Child:  Ngo   uti-c = ma.
         prox  extract-nmz = spec
         Unscrewing (it), here. [Describing herself unscrewing it; adult form would be *usi-ha-t*, as in the mother’s initial utterance.]
  Mother: Aiyi!  Nungon = ta  usi-ha-rok?
         ij   what = ben   extract-pres.sg-2sg
         Aiyi! Why are you unscrewing (it)? (TO, 2;7)

In the initial utterance of the sequence in (10), TO’s mother models the adult-like inflected verb *usi-ha-t* ‘I extract/unscrew’; this is echoed by TO in the following utterances (including two more omitted here to save space) as the nominalized form *usi-k* (which she produces as *uti-c*, where <c > represents a glottal stop), followed by the specifiying enclitic *= ma.* In the final utterance of the sequence, her mother again models the adult-like inflected form of *usi-* ‘extract/unscrew,’ this time inflected for a 2sg subject.

In Sarvasy (2019), I called the period in which TO used such nominalized verb forms with high frequency her ‘root nominal’ stage, in a nod to the ‘root infinitive’ notion (Rizzi, 1993). TO’s use of the nominalized verb form where an adult would use an inflected verb increased dramatically and then decreased to minimal use again between 2;5 and 3;1 (see Table 2, below), but before and during this period, these forms exist in her speech alongside fully inflected verb forms for a range of lexical verbs and inflections.

**Table 2:** TO’s tensed verbs, root nominals, 2sg imperatives, and baby talk verb tokens as percentages of total verb tokens; also, token numbers for the five tenses.

Age	Total verbs	Percent tensed verbs	Percent root nominals	Percent 2sg imperative	Percent baby talk verbs	Tense inflection token numbers				
						Near past	Remote past	Remote future	Near future	Present
2;1	26	0%	0%	31%	0%	0	0	0	0	0
2;2	37	11%	0%	24%	24%	1	0	0	0	3
2;3	78	5%	3%	31%	4%	0	2	0	0	2
2;4	54	15%	2%	35%	4%	5	0	0	0	3
2;5	97	6%	4%	40%	4%	0	1	1	0	4
2;6	77	3%	5%	53%	4%	0	2	0	0	0
2;7	57	7%	32%	47%	2%	1	2	1	0	0
2;8	79	8%	28%	15%	33%	2	2	0	0	2
2;9	92	12%	18%	43%	22%	1	6	0	0	4
2;10	87	11%	14%	33%	9%	1	3	2	0	4
2;11	66	39%	2%	8%	3%	2	6	0	2	15
3;0	205	23%	12%	27%	2%	6	3	0	15	17
3;1	256	43%	6%	14%	0%	16	2	2	57	32
3;2	188	39%	1%	8%	1%	12	5	3	22	32
3;3	324	36%	1%	12%	2%	20	28	0	22	37

As for verb use in context (multi-verb predicates and clause chains), TO’s early clause chains and multi-verb predicates were investigated alongside those of a slightly older child, NN (Sarvasy, 2020, 2021). Her clause chain data contributed to Sarvasy & Choi’s finding (2020b) that children learning clause chaining languages, including Nungon, always produce two-clause chains before producing longer chains of three or more clauses. TO’s multi-verb predicate use emerged at about the same age as clause chain use (2,4–2;5), and she showed upticks in proportional use of both complex structures from the same age (3,1; Sarvasy, 2021).

This brief research report seeks to understand very early Nungon verb productions and investigates the timing of production of the near future tense relative to other verbal inflections, in an expanded sample of four children. It also explores the universality of a ‘root nominal’ stage and of the special CDS ‘do’ auxiliary expansion.

## 2. Methods

The Nungon Child Speech Corpus (NCSC) contains approximately 180 h of digital transcriptions and audio and video recordings of interaction sessions between each of nine target children acquiring the Towet dialect of Nungon as a first language, and one or more caregivers and other family members. In most sessions, a Zoom H5 recorder and a Canon video camera were set up on small tripods by local research assistants, all classificatory kin of the children, and then left in place during the recording session. Parents were informed that the purpose of recording sessions was to elicit speech from the target children through joint play, conversation, and other mostly-sedentary activities, which they could choose themselves during the session, according to how the child responded. During sessions, parents and children looked at photo books containing photos of community members and visitors and an ethnobotanical field guide to the area; reminisced; joked; cooked or pretended to cook; constructed model buildings; and called out to others passing by, among other activities. (A small group of plastic toys including an airplane and figurines were also supplied, but research assistants found early on that these mostly elicited silent, nonverbal play from the children or just discussion of whether a similar one would be procured for the child, rather than verbal imaginative play, and so they were rarely used). When all else failed, parents sometimes engaged in verbatim narrative prompting (Sarvasy, 2023), in which they fed children personal experience narratives from the child’s perspective, to be repeated by the child, clause by clause. All children were selected based on their ages and the criteria that they must live in Towet village and have parents who were both native speakers of the Towet village Nungon dialect.

The corpus was built in three instalments. First, between 2015 and 2017, five target children were recorded for 1 h monthly for 17–24 months. These target children were aged 1;1, 2;1, 2;10, 3;5, and 3;8 at the beginning of the study; these were selected by local research assistants based on birthdates, with the aim of obtaining a cohort with initial ages as close as possible to 2;0, 2;6, 3;0, and 3;6, to be comparable to the Ku Waru longitudinal corpus (Rumsey et al., 2020); the youngest child was added later, to contribute data on early speech production. In 2019, three additional children were tracked in a denser and shorter-term study: each child was recorded in about four one-hour sessions during the course of 1 week, each month, for 5 months, beginning at age 2;4 (two children) and 2;7 (one child); these children were selected to be between 2;0 and 3;0, since the first cohort had shown that this was the period when inflectional morphology development was evident. Finally, a ninth child’s language development was documented in another short-term study in 2023; the child, aged 1;7 at the study’s outset, was recorded for four 30-min sessions during 1 week per month, over 3 months, to try to obtain additional information about very early speech production. Transcriptions and audio/video recordings from this last study had not yet been brought to the city to be transmitted to me for further analysis by the time of the writing of this report.

To investigate early verb productions, I extracted the earliest transcriptions for the four youngest children of the first eight target children in the NCSC. These were for ages 1;1–2;7 for AB, 2;4–2;8 for MK and MF, and 2;1–3;3 for TO. TO and her parents’ utterances were already hand-coded for all verbal inflections and constructions between ages 2;1 and 3;3, from previous case studies of her and her parents’ verb productions (Sarvasy, 2019, 2020, 2021). For this comparative report, I also hand-coded all of the children’s spontaneous utterances (excluding [self-]repetitions), and all of their parents’ ‘do’ auxiliary utterances, for: the boy AB, recorded for an hour monthly from ages 1;1 through 2;7, and for the children MK and MF, recorded for 1.5 to 4 hours in 1 week per month at ages 2;4 through 2;7 (Since MF already produced a range of advanced verbal constructions at ages 2;4–2;5, I did not code her sessions beyond 2;5; since MK already used a wide range of verbal inflections and complex sentence constructions in a dense sample by age 2;7, and since AB’s data only go up to 2;7, I also did not code MK’s productions in her final four sessions at 2;8.)

In the comparative discussion that follows, children’s ages are rounded up or down to the nearest month. For instance, AB was recorded at 19.1 months and then again at 19.6 months; the second recording is discussed here as representing age 1;8. His recording at age 25.7 months is taken to represent speech at 2;2.

## 3. Results

Only the data from AB elucidate very early verb productions. AB uses no verbs, even non-inflecting baby talk lexical verbs, from age 1;1 through 1;4. AB’s data then indicate an extended period of use of just non-inflecting ‘baby talk’ verb forms and 2sg imperatives from 1;5 through 1;7; AB then uses both 2sg and 1sg imperatives, along with baby talk verbs, from 1;8 through 1;9; these are followed by the present tense and 1pl imperatives, then by other tense inflections, aspectual constructions, and dependent verb forms in a rapid expansion of verb forms by age 2;3, when he also produces his first two-clause chains. AB produces his first near future tokens in the session at age 2;4, and his first three-clause chain at 2;5.

TO’s data begin at age 2;1, and in that session, her verb productions are restricted to uninflected baby talk forms and 2sg imperatives, reminiscent of AB’s speech at about ages 1;5–1;7. At 2;2, she expands on this with the addition of 1pl imperatives and present tense forms. Additional tense, imperative, and aspectual constructions are added to the mix in each subsequent session; by age 2;5, TO has produced tokens of four tense inflections, both imperatives, and some dependent verb forms. Her first two-clause chains occur at age 2;5.

TO and AB’s early verb inflections appear to follow a cross-linguistically common phenomenon by which imperative forms are among the first produced by children (Aikhenvald, 2010: 325–330; Aksu-Koç and Ketrez, 2003: 39). Overall, between the ages of 2;1 and 2;5, TO appears to pass through similar stages to AB, but at slightly older ages. At 2;3, however, TO also begins to produce ‘root nominals’: nominalized verbs used in lieu of a tense- or mood-inflected verb; these root nominals increase dramatically and gradually decrease in proportion to TO’s other verb types between 2;5 and 3;1, peaking at 32% of all verb tokens (with multiple types represented) in a session. This is shown in Table 2, alongside TO’s other verb types.

Table 2 also shows the striking development of the near future tense in TO’s speech. TO never produces the near future tense until age 2;11, but then from age 3;0 on, she produces more near future tokens than tokens of any other tense in all sessions except at 3;3, when the near future tense is second in number of tokens to the remote past tense. TO’s verb inflections between 2;1 and 2;7 are compared to the other children’s in Tables 3, 4 (which explores just tokens and types of the near future tense).

**Table 3:** Verb token numbers and verb inflections used per age for the four children.

	TO		AB		MK		MF	
	Verb tokens	Verb inflections	Verb tokens	Verb Inflections	Verb tokens	Verb inflections	Verb tokens	Verb inflections
1;7	–	–	12	imp, bt	–	–	–	–
1;8	–	–	10	imp, bt	–	–	–	–
1;9	–	–	16	imp, bt	–	–	–	–
1;10	–	–	–	–	–	–	–	–
1;11	–	–	–	–	–	–	–	–
2;0	–	–	10	imp, bt, pres	–	–	–	–
2;1	26	imp	6	imp, bt, pres	–	–	–	–
2;2	37	imp, bt, pres, np	5	imp, np, dep	–	–	–	–
2;3	78	imp, bt, delimp, pres, rp, nmz	41	imp, delimp, pres, np, rp, dep, cont, desid	–	–	–	–
2;4	54	imp, bt, delimp, pres, np, dep, nmz	33	imp, pres, nf, dep, proh	28, 23, 27	imp, pres, np, dep	50, 78, 68, 174	imp, delimp, pres, nf, np, rp, irr, dep, desid, cont, compl, proh, infr
2;5	97	imp, bt, pres, rf, rp, dep, nmz	63	imp, pres, nf, dep, cont, proh	78, 48, 35, 18	imp, delimp, pres, np, rp, irr, dep, desid, proh, nmz	192, 37	imp, delimp, pres, nf, np, rp, dep, desid, cont, hab, proh
2;6	77	imp, bt, rp, dep, nmz	98	imp, bt, pres, nf, np, rp, hab, nmz	61, 44, 56, 43	imp, delimp, pres, nf, np, rf, rp, dep, hab, cont, desid, proh, prob	–	–
2;7	57	imp, bt, np, rf, rp, dep, nmz	51	imp, pres, nf, np, rp, dep, desid	120, 128, 111, 129	bt, imp, delimp, pres, nf, np, rf, rp, dep, cont, hab, proh, prob	–	–

**Table 4:** Comparison of near future and baby talk verb tokens and types per one-hour session.

	Near future tokens (types)				Baby talk verb tokens (types)			
Age	TO	AB	MK	MF	TO	AB	MK	MF
2;3	0	0	–	–	3 (2)	0	–	–
2;4	0	2 (2)	0; 0; 0	0; 2 (2); 0; 11 (8)	2 (1)	0	0; 0; 0	0; 0; 0; 0
2;5	0	3 (3)	0^1^; 0; 1 (1); 0	7(7); 0^2^	4 (1)	0	0; 0; 0; 0	0; 0^2^
2;6	0	8 (5)	3 (3); 3 (3); 1 (1); 1 (1)	–	3 (1)	2(1)	0; 0; 0; 0	–
2;7	0	3 (3)	9 (7); 8 (4); 4 (4); 6 (5)	–	1 (1)	0	1 (1); 0; 0; 0	–

^1^This session included two instances of repetitions by MK of parental near future tokens, plus one disfluency that may have been an attempted near future production by MK.

^2^At 2;5, the second session for MF was about 30 min, instead of 1 h, long.

In contrast to TO, as seen in Table 4, AB produces two near future tokens (of two different verbs) at age 2;4, and then three tokens at age 2;5, followed by eight at 2;6, then three at 2;7. Thus, AB shows no evidence of a protracted gap between the onset of production of other tenses and the onset of production of the near future tense, unlike TO. Also in marked contrast to TO, AB shows little evidence of a major ‘root nominal’ stage. In just one session, at 2;6, AB produces four tokens (two types) of nominalizations as main verbs, but these differ from the root nominals used by TO in that AB’s nominalizations involve reduplication, which is the most common form of nominalization in standard Nungon, and is not the form used in the ‘do’ auxiliary construction.

Across all his sessions, AB produces only two ‘do’ auxiliary constructions, shown in (11) and (12):

11. Nan    hoit   ta-a-k.
     Father   hold.bt.nmz do-pres-3sg
     Father does holding (it). [For ‘Father holds it’] (AB, 2;3)

12. Hi-k   to-wangka-t.
     put-nmz  do-nf.sg-1sg
     I’ll do putting (it). [For ‘I’ll put it’] (AB, 2;6)

AB is able to produce the singleton nominalization in the context of the CDS/CS ‘do’ auxiliary construction, but he does not use this nominalization form alone as a main predicate, as TO does. See below for data on AB’s parents’ use of ‘do’ auxiliaries and root nominals.

Overall, TO’s and AB’s early verb productions are similar, with both relying heavily on 2sg imperative forms and, to a lesser extent, uninflecting baby talk forms, with rapid expansion, over a few months, to production of a much wider range of verb forms by about 2;4–2;5. Both children begin to produce complex sentences (clause chains) at the same time as they show the ability to produce a wide range of verbal inflections (at 2;3 for AB and at 2;5 for TO). But TO displays two idiosyncratic patterns that are not shared by AB: a ‘root nominal’ stage, and a marked delay in production of the near future tense inflection.

MK and MF belong to a second cohort of children, studied over a shorter period but with more data collection per month. Three one-hour-long recording sessions were made with MK at age 2;4, and four one-hour-long recording sessions were made at 2;5, 2;6, and 2;7. For MF, four one-hour recording sessions were made at 2;4, and one one-hour and one thirty-minute recording session were made at 2;5.

At age 2;4, MF exhibits an advanced level of speech production, relative to the three other children. In this first month, she already uses two aspectual inflections that never occur in the others’ speech in the study period: the ‘inferred imperfective’ (nine tokens, three types), as in (13), and completive (three tokens, one type), as in (14). In two of her one-hour sessions, she produces more verbs than the other three children do in any of their sessions through 2;7 (verbs per utterance is one proxy for early language development in Nungon: Sarvasy, 2019).

13. xxx  ngo  eto-nga   ta-ga-mok,   ngo.
       prox  forget-mv.ss do-infr-1du prox
    We two seem to be forgetting this one, here. (MF, 2;4)

14. Nok urop  yo-ng = dup.
     1sg  enough  say-dep = compl
     I (have) already said it all. (MF, 2;4)

MF shows no evidence at all of root nominals.

MK’s three one-hour recording sessions at age 2;4 show an apparent paradox: syntactically, her utterances can be relatively advanced, often involving two-clause chains and even one three-clause chain (which TO only begins to produce at age 3;1, and AB at 2;5), but the number of different verbal inflections she produces are confined to: dependent, ‘medial’ verb forms (both different- and same-subject), imperative forms (1sg, 1du, 1pl, and 2sg), a handful of present tense verbs, and just one near past tense verb token. Could it be that, unlike TO and AB, MK mastered complex syntax before producing a range of different verb inflections? In the 4 h of recordings at age 2;5, MK continues to produce two-clause chains and a range of dependent verb inflections within them, along with mostly imperatives (1sg, 1du, 1pl, 2sg and 3sg) and some present tense verbs. She also shows relatively frequent use of the desiderative construction, which she uses from one to three times per session with a variety of lexical verbs. At age 2;6, MK produces a wider range of tense inflections, including increased use of the near future, and tokens of the remote future and remote past, along with the present and near past.

In sum, the four children’s verb production data suggest that each child has a slightly different approach to early verb production. TO shows a reliance on root nominals, and delayed production of the near future. AB produces a wide range of inflections at 2;3–2;4, when he begins to produce complex sentences, but never produces the remote future tense in the study period. MK produces almost entirely dependent verbs in clause chains and imperatives in three sessions at 2;4, and still relies heavily on imperatives at 2;5. Only at 2;6 and 2;7 does she use a wider range of verb inflections in recording sessions. MF is already more advanced than the other three children at age 2;4, producing more verb tokens per session overall, and using both complex syntax and a wider range of verb forms and constructions, two of which are never produced by the other children in this period.

Clearly, sampling and context play a role in the production of some forms–especially rarer ones, like the remote future tense. AB and MF never produce the remote future tense in the coded sessions. At 2;6, MK produces just one remote future token each in two of four sessions. The importance of a dense sample can be seen for baby talk verbs and the near future tense (Table 4) for MK and MF: some sessions at ages 2;4–2;5 lack the near future tense entirely, and MF produces baby talk verbs in only one session out of six in the study period. Overall, it appears that some forms, like the remote future tense, are less likely to be used by children in these sessions (even though TO produces this before she produces the near future tense, she only produces two tokens of it, one each in two different sessions, between 2;1 and 2;8).

Similarly, MK’s reliance on imperatives at 2;4 and 2;5, when she already produces two-clause chains and even a three-clause chain, could be partially related to the recording context (rather than indicative of an early developmental stage). In these early sessions, she produces relatively few utterances containing verbs, and these mostly express her immediate desires through the various imperative forms and, at 2;5, through the desiderative construction. It might be that MK does have command of other verbal inflections at this time, but the artificialness of conversing with one adult for 1 h takes some getting used to; perhaps she is unused to interacting with her father, her primary interlocutor here, for such extended periods. After the initial 2 months of the recording session experience, she begins to discuss other things. This may or may not be related to her linguistic development, as well as her comfort level with the recording setup.

It would be hard, however, to explain TO’s root nominal stage as just an artifact of sampling, since the other children do not use similar root nominals, and TO’s mother appears to ‘fine-tune’ (Bohannon III et al., 1982) her speech to TO’s own idiosyncracies by using root nominals in CDS during the period in which TO herself relies most heavily on these forms (Sarvasy, 2019), but with a lag following TO’s own patterns. The consistent absence of near future forms in all TO’s sessions until 2;11, despite frequent use in CDS in the recording sessions, also suggests that this may not just be a sampling effect.

The data here show that the ‘do’ auxiliary construction is used by all parents who feature in the sessions. It is also produced occasionally by every child, including MF (two tokens/types at 2;4; one at 2;5) and MK (four tokens/types at 2;7). But only TO seems to use nominalized verb forms both in ‘do’ auxiliary constructions and as an optional, ‘root nominal,’ default verb form, without the auxiliary. The reasons for TO’s root nominal use are unclear. AB’s father shows a striking pattern, however, that may help explain the origin of the root nominal stage–not in AB, who lacks this, but in TO.

When AB is 1;5–when he is only just beginning to produce early verbs–his father produces 251 verbs. Of these, 90 (35.8%) are directly inflected verbs, 89 (35.4%) take the CDS ‘do’ auxiliary form, 20 (7.9%) are uninflecting baby talk verbs, and 52 (20.7%) are actually root nominals: nominalizations as used in the CDS ‘do’ auxiliary construction, but without an auxiliary verb. These root nominals occur in two contexts. First, AB’s father often first uses a nominalization with an auxiliary, but then immediately afterward, uses it with no auxiliary, as seen in (15).

15. Ö  na-mo-k     to-i    oro,  na-mo-k,
     conj 1sg.o-give-nmz do-imp.2sg good  1sg.o-give-nmz
     na-mo-k.
     1sg.o-give-nmz
     And do giving (it) to me, okay, giving (it) to me, giving (it) to me. (AB 1;5)

Second, AB’s father also uses root nominals without any immediately preceding auxiliary construction, as in (16):

16. Toi-k,   nogo   toi-k.
     arrange-nmz pro.1sg.foc arrange-nmz
     Arranging (it), it’s I, arranging (it). (AB 1;5)

It seems clear that TO’s pattern of using root nominals stems from CDS like (15) and (16). But it remains a puzzle why she seems to seize on these forms in her ‘root nominal stage’, and yet AB, who clearly hears them, does not.

AB’s primary interlocutor in the recording sessions from 1;6 through 2;2 is his mother. Although she does occasionally use the ‘do’ auxiliary construction, she appears to use it in much lower frequencies than does his father in the session at 1;5; for instance, she uses it only once in the session at 1;6. AB’s older sister features in the session at 1;7 (which was cut off due to a technical error), and also uses one ‘do’ auxiliary construction. When AB’s father again serves as his primary interlocutor, at 2;3, his CDS is radically different from the session at 1;5. AB’s father produces 329 verbs at 2;3; none are baby talk verbs or root nominals, and only 6 (1.8%) are ‘do’ auxiliary constructions. Although we cannot know how AB’s father addresses AB in the months between 1;5 and 2;3, it appears that the father’s early heavy use of ‘do’ auxiliaries and root nominals may be phased out some time before AB begins to produce a wide range of verbs in greater numbers–hence, maybe, it is not influential in shaping AB’s own speech. But it could also be that AB’s father does not habitually interact with him as frequently as his mother, who has lower frequencies of ‘do’ auxiliaries than his father even at 1;6.

As shown in Sarvasy (2019), TO’s mother uses the ‘do’ auxiliary construction in almost every session between 2;1 and 3;2, but it is much less frequent in her CDS to TO than it is in AB’s father’s CDS to AB at 1;5: maximally just 7% of all TO’s mother’s verb tokens are framed as ‘do’ auxiliaries (at 2;5: 51 of 721 total verbs), and ‘do’ auxiliaries are an average of 3% of her verbs per session throughout the study period. This is slightly higher than the percentage in AB’s father’s speech at 2;3. TO’s father uses the ‘do’ auxiliary construction at least once in all seven sessions in which he features between 2;1 and 3;3, but ‘do’ auxiliary tokens average just 1.4% of verbs per session for him. As for root nominals in TO’s parents’ CDS, these occur sparingly between 2;1 and 3;3; they are absent from some sessions, and never reach 1% of all verb tokens–again, in contrast to AB’s father’s CDS at 1;5.

In sum, AB’s father’s early CDS shows clearly how a child might learn to use root nominals from repetitions of just the nominalized part of the CDS ‘do’ auxiliary construction (as in 15), and from root nominals in CDS (as in 16). AB’s father’s early CDS has very high proportions of ‘do’ auxiliaries and root nominals relative to inflected verbs, but these are much lower in AB’s mother’s CDS. We have no data on TO’s parents’ early CDS; at the beginning of the study period for TO, at 2;1, their use of ‘do’ auxiliary constructions and root nominals is much less frequent than AB’s father’s use at 1;5. It could be that their slightly higher frequencies of ‘do’ auxiliaries and root nominals in the older period here (2,1 and up) is the key reason that TO goes through a ‘root nominal stage’ and AB does not–but it could also be argued that TO seizes on the root nominals as a preferred form during this stage, and her parents simply respond to this in their CDS (as suggested by Sarvasy, 2019).

Apart from morphosyntax (‘do’ auxiliaries and root nominals), very few obvious un-adult-like ‘errors’ in inflection are evident in the data here. I noted one mismatch in switch-reference use, where what should have been marked as same-subject was marked as different-subject. In some early transcripts, MK appears to possibly use the 1du *ongo-ra!* ‘go-imp.1du,’ ‘let us (two) go!’ and the 1pl *ongo-na! ‘go-*imp.1pl,’ ‘let us (three or more) go!’ interchangeably, such that she might not truly be tracking the number of the subject. But she could be construing ‘we’ differently when she uses the two different forms (either just her and her mother, or her, her mother, and the experimenter), so this is hard to know for sure. Otherwise, there is no evidence that one particular inflection (other than the root nominal for TO) is used as a ‘default’ form by the children.

## 4. Conclusion

This study has shown that four children learning Nungon show some similarities in early verb productions: the early part of the third year of life features increasing numbers of verb tokens, verbal inflection types, and the development of complex syntactic structures involving verbs. But the children show much variance in the order of production of different verbal inflections. TO, in particular, has an entire ‘root nominal’ stage that appears to be absent in the development of the other three children.

Recent years have seen several recommendations for expanding the range of languages for which child acquisition is documented (Pye, 2021, 2022; Kidd and Garcia, 2022; Hellwig et al., 2023). These acknowledge that building searchable hundred-hour corpora requires many hundreds, perhaps thousands, of hours of work. They propose various ways to produce useful and informative documentation of child language acquisition for diverse languages with a reduced corpus size.

One approach is the minimalist ‘acquisition sketch’, described in Defina et al. (2023). Defina et al. (2023) propose a minimum sample of 5 h of processed data, with two children whose speech is sampled at six-month intervals, beginning at age 2;0 and ending at age 4;0. This sample will surely be useful for gaining an overall understanding of the general trajectory of language acquisition, but it would not be useful for detailed study of the acquisition of verbal inflections, as seen here, due to the time lapse between samples. Further, the picture that emerged in the current study, in which TO’s root nominal stage and delayed near future tense are outlier features, would not be possible to capture with just two of these children: either TO would be omitted from the sample, and her idiosyncracies absent from an acquisition account, or it would be hard to know who was the outlier: TO or the other included child.

As anticipated by Rowland and Fletcher (2006), denser corpora enable the capture of rarer verbal inflections, like the remote future tense in these data. One downside to denser corpora, however, may be evident in the content of later recording sessions within each data collection week, at least in MK’s dataset. Impressionistically, MK seems to protest more and ask to leave the recording session more in the third and fourth session of each month; at ages 2;5 and 2;6, the first recording session contains the most verbs of any session of the collection week. Four one-hour sessions in 1 week could be just too much for her at those ages (in contrast, MF’s fourth recording session in a week at age 2;4 is her most verbose: so this is not true for both children in the dense collection cohort).

This report is a beginning step toward understanding early verbal inflection production in Nungon. Future work should expand on this by quantifying type/token productions, attempting to classify any production ‘errors’ according to frequency in the corpus (following Aguado-Orea and Pine, 2015 and others), and investigating in more detail, possibly even through day-long recordings, the relationship between verbal inflections in CS and CDS.

## Data availability statement

The data analyzed in this study is subject to the following licenses/restrictions: available to other researchers by contacting the author; a small portion are freely available on CHILDES. Requests to access these datasets should be directed to HS, h.sarvasy@westernsydney.edu.au.

## Ethics statement

The studies involving humans were approved by the Australian National University Ethics Committee; Western Sydney University Human Ethics Committee. The studies were conducted in accordance with the local legislation and institutional requirements. Written informed consent for participation in this study was provided by the participants’ legal guardians/next of kin.

## Author contributions

HS coded the data, analyzed them, and wrote the paper.

## Funding

This work was supported by the Australian Research Council, grants CE140100041 and DE180101609.

## Conflict of interest

The author declares that the research was conducted in the absence of any commercial or financial relationships that could be construed as a potential conflict of interest.

## Publisher’s note

All claims expressed in this article are solely those of the authors and do not necessarily represent those of their affiliated organizations, or those of the publisher, the editors and the reviewers. Any product that may be evaluated in this article, or claim that may be made by its manufacturer, is not guaranteed or endorsed by the publisher.

## Figures and Tables

**Table:** 

…	Omitted content
1sg, 2du, etc.	Person and number
adv	Adverb
bt	Baby talk form
compl	Completive
cont	Continuous
delimp	Delayed imperative
dep	Dependent
desid	Desiderative
ds	Different-subject
foc	Focus
hab	Habitual
ij	Interjection
imp	Immediate imperative
infr	Inferred imperfective
loc	Locative
mv	Medial verb
near	Nearby
nf	Near future
nmz	Nominalization
np	Near past
pres	Present
prob	Probable
proh	Prohibitive
prox	Proximal
rf	Remote future
rp	Remote past
spec	Specifier
ss	Same-subject
top	Topic
xxx	Unintelligible
